# O03 Are proposed definitions of low-risk *Staphylococcus aureus* bacteraemia (SAB) clinically useful?

**DOI:** 10.1093/jacamr/dlae217.003

**Published:** 2025-01-23

**Authors:** Karla Berry, Simon Dewar, Heather W Dolby, George Cooper, Rebecca K Sutherland, Clark D Russell

**Affiliations:** Clinical Infection Research Group, Western General Hospital, Edinburgh, Scotland; Clinical Infection Research Group, Western General Hospital, Edinburgh, Scotland; Centre for Inflammation Research, Institute for Regeneration and Repair, The University of Edinburgh, Scotland; Centre for Inflammation Research, Institute for Regeneration and Repair, The University of Edinburgh, Scotland; Clinical Infection Research Group, Western General Hospital, Edinburgh, Scotland; Centre for Inflammation Research, Institute for Regeneration and Repair, The University of Edinburgh, Scotland

## Abstract

**Background:**

Identifying ‘low-risk’ patients with *Staphylococcus aureus* bacteraemia (SAB) could enable earlier switches to oral antibiotics and reduce the need for intensive investigation in selected patients. Several definitions have been proposed but have not been compared with each other.

**Objectives:**

To determine the agreement between current definitions of ‘low-risk’ SAB.

**Methods:**

We studied 600 patients included in an ongoing retrospective cohort study of consecutive adults with SAB from three hospitals in South East Scotland, identified between 20/12/2019 and 23/4/2023. We investigated four definitions of ‘low-risk’ SAB: (1) patients meeting eligibility criteria for the SABATO early oral switch trial; (2) patients meeting a definition of low-risk SAB recently proposed by Hendriks *et al*. (*Clin Infect Dis* 2024; **79**: 43–51); (3) patients meeting the eligibility criteria for IV to oral switch on day 7 in the SNAP trial; and (4) patients not meeting the IDSA definition of complicated SAB. We determined which patients met these definitions, quantified the overlap, determined the agreement between definitions using Cohen’s kappa statistic (*k*), and compared patient characteristics and outcomes between the groups.

**Results:**

A total of 98/600 (16.3%) patients met the SABATO definition, 113/600 (18.8%) met the Hendriks *et al.* definition, 144 (24.0%) met the SNAP early oral switch criteria, and 263/600 (43.8%) did not meet the IDSA criteria for complicated SAB. We observed limited overlap between these groups. Only 29 patients met all four definitions. Agreement between definitions was fair to moderate (*k*=0.21 to 0.46). Patients meeting these definitions shared some characteristics, including age (median 64–67 years), a predominance of nosocomial acquisition and IV source of bacteraemia, and reduced frequency of an unknown source of bacteraemia. In-hospital attributable mortality differed between groups: 4.1% in those meeting the SABATO definition, 10.6% in those meeting the Hendriks et al definition, 3.5% in those meeting criteria for SNAP early oral switch, and 17.1% in those not meeting the IDSA definition for complicated SAB (compared with 24.0% in patients not meeting any definition). Rates of persistent or recurrent bacteraemia also varied: 3.1% for SABATO, 0% for Hendriks et al, 2.8% for SNAP early oral switch, and 0.8% for IDSA non-complicated SAB (compared with 6.4% for patients not meeting any definition).

**Conclusions:**

Given these discrepancies in overlap, agreement, and outcomes, our findings highlight the ongoing need for a standardized definition of ‘low-risk’ SAB to consistently identify these patients in clinical practice and for clinical trials.

